# Analysis of long noncoding RNA and mRNA using RNA sequencing during the differentiation of intramuscular preadipocytes in chicken

**DOI:** 10.1371/journal.pone.0172389

**Published:** 2017-02-15

**Authors:** Tao Zhang, Xiangqian Zhang, Kunpeng Han, Genxi Zhang, Jinyu Wang, Kaizhou Xie, Qian Xue, Xiaomei Fan

**Affiliations:** 1 College of Animal Science and Technology, Yangzhou University, Yangzhou, Jiangsu, China; 2 Key Laboratory for Animal Genetics, Breeding, Reproduction and Molecular Design of Jiangsu Province, Yangzhou, Jiangsu, China; 3 Vazyme Biotech Co.,Ltd., Economic and Technological Development Zone, Nanjing, Jiangsu, China; Universitat de Lleida, SPAIN

## Abstract

Long noncoding RNAs (lncRNAs) regulate metabolic tissue development and function, including adipogenesis. However, little is known about the function and profile of lncRNAs in intramuscular preadipocyte differentiation in chicken. Here, we identified lncRNAs in chicken intramuscular preadipocytes at different differentiation stages using RNA sequencing. A total of 1,311,382,604 clean reads and 25,435 lncRNAs were obtained from 12 samples. In total, 7,433 differentially expressed genes (4,698 lncRNAs and 2,735 mRNAs) were identified by pairwise comparison. These 7,433 differentially expressed genes were grouped into 11 clusters based on their expression patterns by K-means clustering. Using Weighted Gene Coexpression Network Analysis, we identified four stage-specific modules positively related to I0, I2, I4, and I6 stages and two stage-specific modules negatively related to I0 and I2 stages, respectively. Many well-known and novel pathways associated with intramuscular preadipocyte differentiation were identified. We also identified hub genes in each stage-specific module and visualized them in Cytoscape. Our analysis revealed many highly-connected genes, including XLOC_058593, *BMP3*, *MYOD1*, and *LAMP3*. This study provides a valuable resource for chicken lncRNA study and improves our understanding of the biology of preadipocyte differentiation in chicken.

## Introduction

Because of its characteristics of high protein, low cholesterol, and low calorie, chicken is generally accepted as a high-quality source of protein by consumers around the world. The broiler has many advantages over pig and cattle, including a short raising period, a high feed conversion rate, and a high degree of automation in raising and processing. With the improvement of living standards, people in China are paying more attention to meat quality. However, the quality and flavor of chicken have decreased in recent decades as a result of genetic selection for faster growth and increased feed conversion efficiency, particularly in China and many Southeast Asian countries [1].

Studies have shown that intramuscular fat (IMF) content is associated with multiple meat quality characteristics, such as tenderness, juiciness, flavor, and water holding capacity, in chicken, pork, and beef [1–5]. The IMF level is determined mainly by the hyperplasia and hypertrophy of adipocytes distributed among muscle fibers [6]. Adipogenesis is a complex process regulated by various transcriptional events. In mammals, the differentiation of intramuscular preadipocytes has been well studied, especially in bovine and porcine. Previous studies identified *FTO* [5], *GPR39* [7], myostatin [6], microRNA-143 [8], *PPARG* [9], *FABP4* [10], and *LIPE* [11] as key roles in the regulation of intramuscular preadipocyte differentiation and intramuscular fat deposition in pig and cattle. However, the mechanism by which intramuscular fat deposition is regulated in chicken is poorly understood. To date, only a few genes have been identified to be associated with intramuscular fat deposition, such as *H-FABP* [12], *A-FABP* [13], *FAT/CD36* [14], and adiponectin [15].

RNA sequencing (RNA-seq) is a revolutionary tool to identify differentially expressed genes (DEGs) regulating various biological processes. It enables us to discover new genes and therefore to describe unannotated transcriptional activity by identifying numerous noncoding transcripts [16]. Long noncoding RNA (lncRNA), which plays important roles in epigenetic regulation, chromatin modification, genomic imprinting, transcriptional control, and pre-/post-translational mRNA processing [17], has attracted substantial attention in the last few years. Three transcriptomic studies have shown that lncRNAs affect the differentiation of abdominal and subcutaneous preadipocytes [18–20]. However, those studies mainly focused on abdominal and subcutaneous preadipocytes, and little is known about the function and significance of lncRNAs in the differentiation of intramuscular preadipocytes.

In the present study, we investigated the expression profiles of lncRNAs and mRNAs of intramuscular preadipocytes on days 0, 2, 4, and 6 of differentiation by RNA-seq in Jinghai Yellow chicken. Our study aimed to characterize the features of lncRNAs and identify differentially expressed lncRNAs and mRNAs by comparing the transcriptomic profiles of preadipocytes among different stages of differentiation. We also focused on determining the biological processes and pathways that exhibited significant changes in activity during the differentiation of intramuscular preadipocytes. Our work provides a valuable resource for chicken lncRNA study and improves our understanding of the biology of intramuscular preadipocyte differentiation in chicken.

## Materials and methods

### Ethics statement

All animal experiments were reviewed and approved by the Institutional Animal Care and Use Committee of Yangzhou University. This study was approved by Animal Ethics Committee of Yangzhou University

### Primary culture of chicken intramuscular preadipocytes

Chicken preadipocytes from breast muscle tissue were cultured in accordance with the method described by Yuan [21], with some modifications. Jinghai Yellow chicken was provided by Jiangsu Jinghai poultry industry group co., LTD. Breast muscle tissue was collected from 14-day-old Jinghai Yellow chicken under sterile conditions. This tissue was washed using phosphate-buffered saline supplemented with penicillin (100 units/ml) and streptomycin (100 μg/ml). The washed tissue was cut into 1 mm^3^ pieces using surgical scissors and then digested using 2 mg/ml collagenase type II (Sangon Biotech, Shanghai, China) with shaking for 2 h at 37°C. The digested cell suspension was filtrated using 200- and 400-mesh screens and centrifuged at 1500 rpm for 10 min at 22°C to separate the stromal–vascular fraction from undigested tissue debris and mature adipocytes. Stromal–vascular cells were plated onto a 60-mm culture plate at a density of 1×10^5^ cells/ml and cultured with Dulbecco’s modified Eagle’s medium/Ham’s nutrient mixture F-12 (DMEM/F12) basic medium [10% (v/v) FBS, 100 units/ml penicillin, and 100 μg/ml streptomycin] in a humidified atmosphere with 5% (v/v) CO_2_ at 37°C until reaching 90% confluence.

### Induction of differentiation

**Fig 1:**
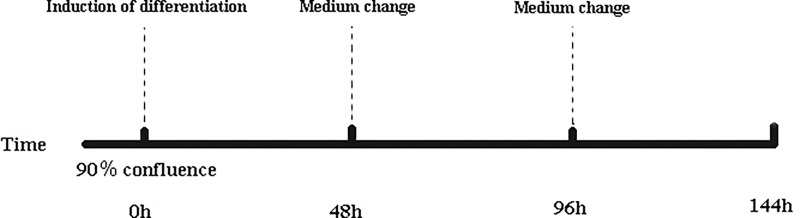
Procedure for inducing the differentiation of intramuscular preadipocytes. Basic medium consisted of DMEM/F12 and 10% FBS. Differentiation induction medium consisted of basic medium, insulin, dexamethasone, and IBMX. Maintenance medium consisted of basic medium and insulin. The differentiation induction medium was replaced with the maintenance medium at 48 h, which was in turn replaced with the basic medium at 96 h.

After reaching 90% cell confluence, the basic medium was removed and replaced with differentiation medium [0.25 μM dexamethasone (Takara, Dalian, China), 10 μg/ml insulin (Takara), and 0.5 mM IBMX (Takara)] for 48 h. Then, the differentiation medium was replaced with maintenance medium [10 μg/ml insulin (Takara)] and incubated for 48 h. The detailed procedure for the induction of intramuscular preadipocytes is described in Fig 1. Cells were collected 0, 48, 96, and 144 h after induction (referred to as stages I0, I2, I4, and I6, respectively). Each stage included three biological replicates (n = 3).

### RNA extraction, library construction, and sequencing

A total of 12 cell samples were successfully collected. Total RNA was extracted using Trizol reagent (Invitrogen). The integrity, concentration, and purity of total RNA were checked using Nanodrop, Qubit 2.0, and Agilent 2100. RNA samples with an RNA integrity number greater than 8.0 and an optical density 260/280-nm ratio greater than 1.9 were selected for deep sequencing. The rRNA was removed, and mRNA was enriched using magnetic beads with oligo(dT) and then randomly fragmented using fragmentation buffer. The mRNA was used as a template to synthesize the first-strand cDNA using 1st Strand Enzyme Mix (Vazyme). The second-strand cDNA was synthesized using 2nd Strand Marking Buffer and 2nd Strand/End Repair Enzyme Mix (Vazyme). The products were purified by VAHTSTM DNA Clean Beads and then the end of the double strand was repaired and A-tailed. An adapter was joined to the A-tailed products using ligation mix. Suitably sized fragments were selected using VAHTSTM DNA Clean Beads to construct the cDNA library by PCR. The RNA sequencing was performed using Illumina HiSeqXTen by Vazyme Biotech Co., Ltd. The sequencing data were submitted to the National Center for Biotechnology Information Sequence Read Archive under Accession No. SRP080792.

### Quality control

The raw data were subjected to quality control using FastQC (http://www.bioinformatics.babraham.ac.uk/projects/fastqc/). The base composition and quality distribution of reads and the GC and AT base content were analyzed, which could reflect the quality of the raw data as a whole. Clean data were obtained by removing reads containing adapters, reads containing over 10% poly-N, and low-quality reads (>50% of bases whose Q scores were ≤10%) from the raw data.

### Sequencing data analysis

The clean data were mapped to the *Gallus gallus* reference genome (gal4) by the TopHat2 [22] program using the following parameters: segment length, 25; segment mismatches, 2. For the remaining parameters, the default settings were used. The uniformity, insert length, and saturation of sequencing data were analyzed based on the alignment results. The transcripts were assembled using the Cufflinks [22] program based on RABT assembly strategies.

### lncRNA prediction

Based on the assembly results, transcripts with RPKM = 0 were removed. The filter criteria of lncRNAs were as follows: 1) transcripts in the “i,” “j,” “x,” “u,” and “o” classes were included; 2) transcripts shorter than 200 nt were excluded; 3) the open reading frame (ORF) was predicted using TransDecoder (https://transdecoder.github.io/) and transcripts with an ORF that was longer than 300 nt were removed; and 4) Coding-Non-Coding Index (CNCI)[23], Coding Potential Calculator (CPC)[24], Coding-Potential Assessment Tool (CPAT)[25], and Pfam Scan (v1.3) [26] were used to distinguish mRNA from lncRNA. CNCI can effectively distinguish protein-coding and non-coding sequences independent of known annotations by profiling adjoining nucleotide triplets. CPC assesses the protein-coding potential of a transcript based on six biologically meaningful sequence features. CPAT can rapidly recognizes coding and noncoding transcripts from a large pool of candidates using a logistic regression model built with four sequence features: open reading frame size, open reading frame coverage, Fickett TESTCODE statistic and hexamer usage bias. Pfam Scan (v1.3) used to identify occurrence of any of the known protein family domains documented in the Pfam database.

### Prediction and annotation of lncRNA targets

lncRNA functions by acting on protein-coding genes via cis-acting elements and trans-acting factors. In the present study, lncRNA targets were predicted based on cis function prediction. The closest coding genes to lncRNAs 100 kb upstream and downstream of them were screened and their associations with lncRNA were analyzed using the Bedtools program [27]. Some antisense lncRNAs could also regulate gene silencing, transcription, and the stability of mRNA by binding to sense mRNA. Therefore, we searched for lncRNA targets by predicting the complementary binding between antisense lncRNAs and mRNAs using the RNAplex program [28]. Then, we subjected the target genes to functional enrichment analysis using the DAVID database [29].

### Quantitation of gene expression

Cuffdiff [30] was used to calculate the expected number of fragments per kilobase of transcript sequence per million base pairs sequenced (FPKM) of both mRNA and lncRNA in each sample. For biological replicates, transcripts or genes with a q-value <0.05 and fold change ≥2 were defined as genes or lncRNAs that were differentially expressed between the two groups [31].

### Co-expression network analysis

A co-expression network was constructed using the Weighted Gene Co-expression Network Analysis (WGCNA) package [32] in the R environment with the 2,510 differentially expressed genes. The groups of co-expressed genes, termed “modules”, were detected by the dynamic tree cutting method. Then, the stage-specific modules were identified based on the module–trait relationship (correlation between eigengene and traits), as well as the correlation between *gene significance* (GS) and *module membership* (MM) values. Modules with highly correlated GS and MM values (*P*-value <0.05) and module–trait relationships with a correlation coefficient >0.4 were identified as stage-specific modules. Genes with GS >0.4 and MM >0.8 was identified as hub genes in the corresponding module. The connections among the hub genes were visualized in Cytoscape with the top 200 connections of the top 150 genes for each stage-specific module.

### Gene Ontology and Kyoto Encyclopedia of Genes and Genomes analysis

Functional annotation enrichment analyses for Gene Ontology (GO) and Kyoto Encyclopedia of Genes and Genomes (KEGG) were conducted using the KOBAS server[33]. GO terms and pathways with a *P*-value less than 0.05 were considered as significantly enriched.

### Validation of gene expression by qRT-PCR

Primers were designed using Primer-BLAST on the NCBI website (http://www.ncbi.nlm.nih.gov/tools/primer-blast/) (S1 Table). The first cDNA strand was synthesized using the PrimeScript™ RT Master Mix (Perfect Real Time) kit (Takara, Dalian, China), in accordance with the user manual. β-actin was used as a housekeeping gene. Gene expression was quantified using AceQ qPCR SYBR Green Master Mix (Vazyme, Nanjing, China). The 20-μL PCR reaction included 10 μL of AceQ® qPCR SYBR® Green Master Mix (Vazyme, China), 0.4 μL (10 pM/μL) of specific forward primer, 0.4 μL (10 pM/μL) of reverse primer, 0.4 μL of ROX reference dye, 2 μL (10 ng/μL) of diluted cDNA, and 6.8 μL of RNase-free water. Cycling parameters were 95°C for 5 min, followed by 40 cycles of 95°C for 10 s and 60°C for 34 s. Melting curve analyses were performed following amplifications. The ABI 7500 software was used to detect the fluorescent signals. Quantification of selected gene expression was performed using the comparative threshold cycle (2^−ΔΔCT^) method.

## Results

### Sequencing results and quality control

A total of 1,382,347,320 raw reads were produced from the 12 cDNA libraries. After quality control. 1,311,382,604 clean reads (196.71 Gb) were obtained. The proportion of clean reads among the raw reads of the 12 libraries ranged from 92.6% to 96.8%. The proportion of reads with a Phred quality value greater than 30 among the clean reads ranged from 91.94% to 92.97%. The average GC content of the clean reads of the 12 libraries was 54.39%. Overall, 67.20% to 80.10% of the clean reads were aligned against the *Gallus gallus* reference genome. Among these mapped reads, 69.04% to 72.91% of reads were mapped to exon regions, 6.85% to 11.76% of reads were mapped to intron regions, and 17.88% to 20.74% of reads were mapped to intergenic regions (Table 1).

**Table 1:** Statistics of clean reads in intramuscular preadipocytes in chicken.

	I0-1	I0-2	I0-3	I2-1	I2-2	I2-3	I4-1	I4-2	I4-3	I6-1	I6-2	I6-3
Total clean reads	139314960	125290132	135292626	98251064	106968480	97982090	106396072	86563094	88981470	107381816	102101162	116859638
Base number (G)	20.90	18.79	20.29	14.73	16.04	14.70	15.96	12.98	13.35	16.11	15.32	17.53
Q30 reads (%)	92.26	92.57	92.2	92.68	91.94	92.48	92.62	92.28	92.27	92.97	92.5	92.87
Mapped reads	100076318	96475573	103589646	76684304	85728689	74276348	80429295	67366562	59759159	83547437	78726943	91156475
Mapping rate	71.8	77	76.6	78	80.1	75.8	75.6	77.8	67.2	77.8	77.1	78
Exon (%)	75.27	73.55	72.84	72.1	71.41	70.23	69.04	72.91	70.22	72.24	71.95	70.57
Intron (%)	6.85	8.08	8.39	9.58	8.86	9.98	11.76	8.72	10.99	7.98	7.98	8.7
Intergenic (%)	17.88	18.37	18.76	18.32	19.73	19.78	19.2	18.37	18.79	19.78	20.07	20.74

### Identification of lncRNAs in intramuscular preadipocytes

**Fig 2:**
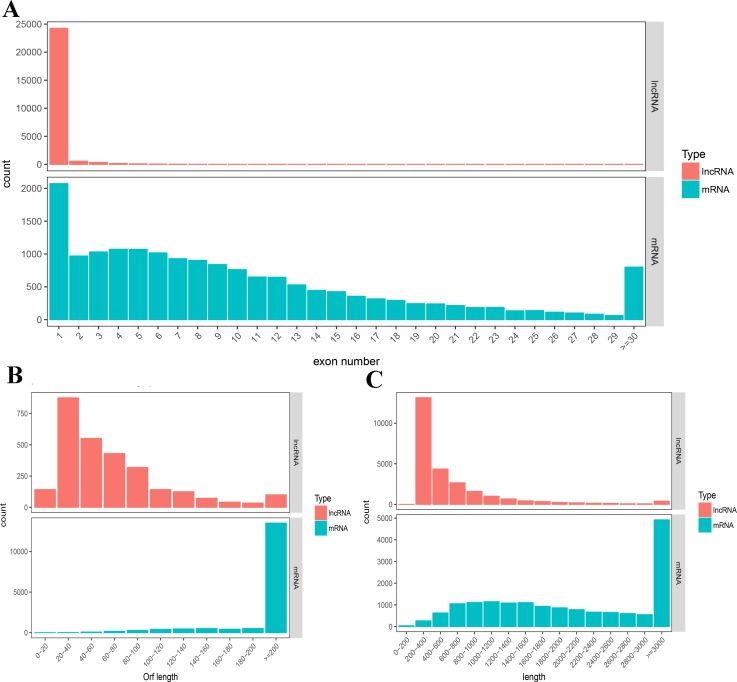
Structure comparison of lncRNAs and mRNAs. A) Distribution of the number of exons of lncRNAs and mRNAs. B) Distribution of the ORF length of lncRNAs and mRNAs. C) Sequence length of lncRNAs and mRNAs.

A total of 25,435 novel lncRNAs were obtained from the 12 intramuscular preadipocyte samples (S1 Appendix). We analyzed the sequence length, ORF length, and exon number of lncRNAs and mRNAs (Fig 2). The results showed that most lncRNAs contained only one exon. The ORF length of the lncRNAs mainly ranged from 20 to 100 bp, while the sequence length was mainly distributed in the range of 200–400 nt. We also found that lncRNAs were shorter in sequence length and ORF length, and had fewer exons than mRNAs in intramuscular preadipocytes.

### Functional prediction of lncRNAs in intramuscular preadipocytes

**Fig 3:**
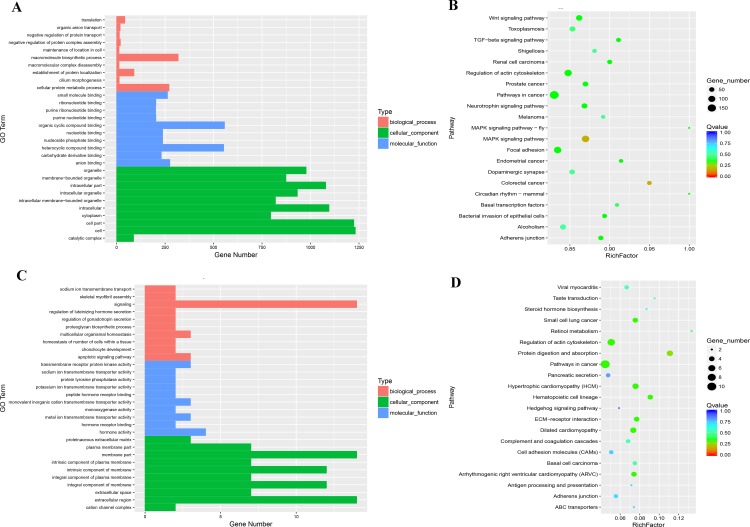
GO and pathway analyses of candidate target genes of lncRNAs in intramuscular preadipocytes.

To explore the functions of the lncRNAs active in intramuscular preadipocytes of chicken, we predicted the candidate target genes of lncRNAs by predicting the cis function between lncRNAs and neighboring mRNAs as well as the complementary binding between antisense lncRNAs and mRNAs. A total of 11,398 target genes were identified for 20,116 lncRNAs by cis function prediction, while 365 target genes were identified for 479 lncRNAs by complementary binding prediction. These 11,398 and 365 candidate target genes were subjected to GO and pathway analyses (Fig 3). GO analysis of the 11,398 genes showed that the macromolecular biosynthetic process, cellular protein metabolic process, and establishment of protein localization were the most abundant terms in the biological process category. In the cellular component category, cell, cell part, and intracellular were the top three terms, while organic cyclic compound binding, heterocyclic compound binding, and anion binding were the most abundant terms in the molecular function category. Pathway analysis indicated that 24 pathways were significantly enriched (*P*<0.05), including the TGF-β signaling pathway, MAPK signaling pathway, and regulation of actin cytoskeleton pathway. The same analysis was performed on the 365 target genes predicted by complementary binding. It was found that signaling, multicellular organismal homeostasis, and apoptotic signaling pathway were the top three terms in the biological process category, and hormone activity, transmembrane receptor protein kinase activity, and metal ion transmembrane transporter activity were the top three terms in the molecular function category, while the equivalent terms in the cellular component category were membrane part, extracellular region, and integral component of membrane. In the pathway analysis, 12 pathways were significantly enriched for the 365 candidate target genes, such as protein digestion and absorption, hypertrophic cardiomyopathy (HCM), and hematopoietic cell lineage.

### Genes differentially expressed during intramuscular preadipocyte differentiation

**Fig 4:**
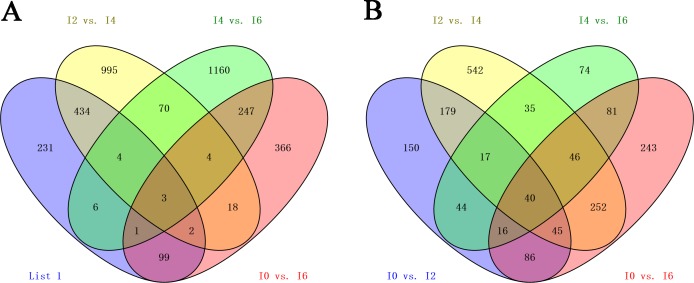
Venn diagram of DEGs at different stages. A) Venn diagram of differentially expressed lncRNAs in four comparisons (I0 vs. I2, I2 vs. I4, I4 vs. I6, and I0 vs. I6). B) Venn diagram of differentially expressed mRNAs in three comparisons (I0 vs. I2, I2 vs. I4, I4 vs. I6, and I0 vs. I6).

Given the criteria of q-value <0.05 and fold change ≥2, 4,698 differentially expressed lncRNAs and 2,735 differentially expressed mRNAs (known protein-coding genes) were obtained by pairwise comparisons (I0 vs. I2, I0 vs. I4, I0 vs. I6, I2 vs. I4, I2 vs. I6, and I4 vs. I6) of samples collected from preadipocytes at days 0, 2, 4, and 6 of differentiation (S2 Appendix). In total, 3,200 differentially expressed lncRNAs and 1,608 differentially expressed mRNAs were obtained by pairwise comparisons (I0 vs. I2, I2 vs. I4, I4 vs. I6) of the same samples. As shown in Fig 4, 43differentially expressed genes were common among four comparisons (3 lncRNAs and 40 mRNAs) (S2 Table).

**Fig 5:**
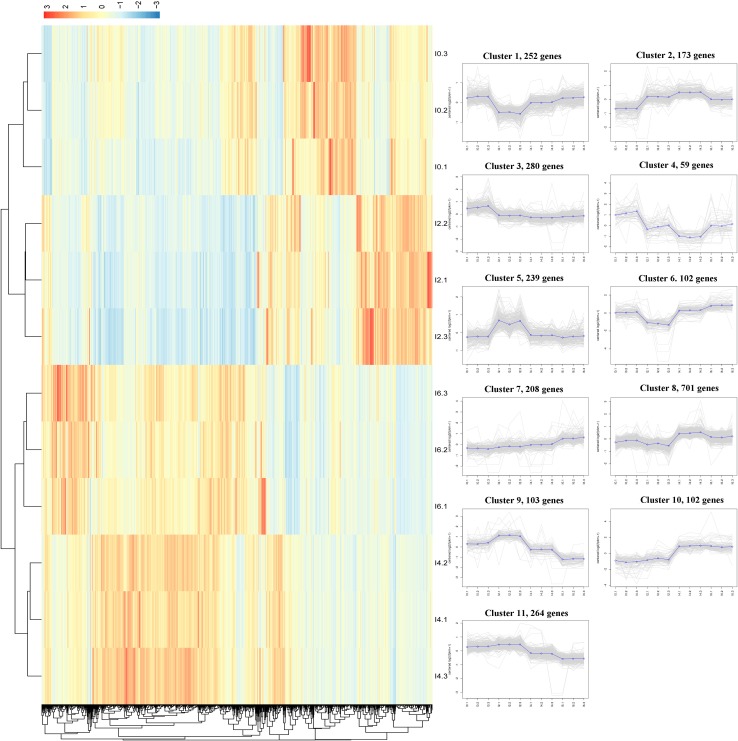
Clustering of all DEGs (lncRNAs and mRNAs). The heatmap shows the K-means clustering of transformed expression values for lncRNAs and mRNAs. Yellow represents higher expression and blue represents lower expression. The line charts represent the expression patterns of genes in 11 clusters corresponding to the heatmap.

We performed K-means clustering of all DEGs using the Euclidean distance method associated with complete linkage (Fig 5, S3 Table). Eleven main clusters were plotted with expression patterns of genes involved. These clusters can be further assigned to six main groups of different dynamic patterns. The first group, including clusters 1 and 6, represents genes for which the expression levels decreased at day 2 and then increased at days 4 and 6 of differentiation. The second group, including cluster 2, represents genes that were upregulated at day 2 and then achieved their highest expression levels at day 4 of differentiation, suggesting that genes in this group play important roles in the differentiation of intramuscular preadipocytes. The third expression pattern, including clusters 3 and 4, represents genes that underwent an overall trend of decrease, suggesting their lack of involvement in intramuscular preadipocyte differentiation. The fourth group, including clusters 5, 9, and 11, represents genes that were significantly upregulated at day 2 of differentiation, suggesting that they are essential in the early stage of intramuscular preadipocyte differentiation. Genes in these clusters included ones known to be important in preadipocyte differentiation, such as *IGFBP2* and *FADS2*. The fifth expression pattern includes cluster 7, which represents genes that maintained relatively constant levels of expression before day 4 and were then significantly upregulated at day 6 of differentiation. This suggests that these genes, including *IGF-1* and *MC5R*, might be involved in the late stage of differentiation of intramuscular preadipocytes. The last group, including clusters 8 and 10, represents genes expressed at lower levels on days 0 and 2, but expressed at significantly higher levels at days 4 and 6 of differentiation. Members of this group such as *BMP6*, *FABP4*, *PPAR*, and *FAT4* are well known to be related to preadipocyte differentiation.

**Fig 6:**
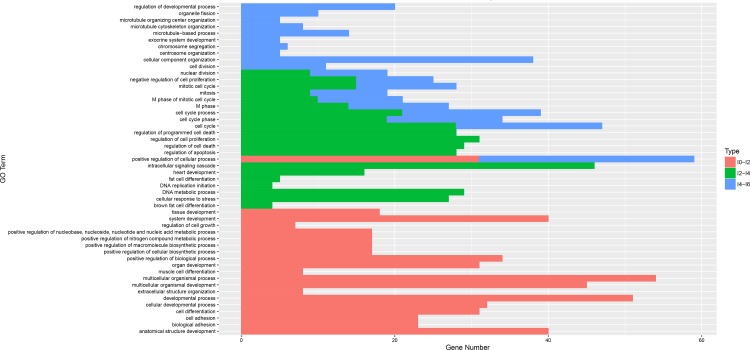
Biological process analysis of DEGs in three comparisons. Red, green, and blue represent the I0–I2, I2–I4, and I4–I6 comparisons, respectively. The individual and overlapping areas in the histogram represent stage-specific and common biological processes between different comparisons.

**Fig 7:**
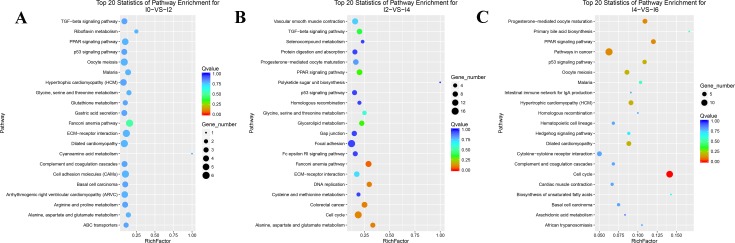
Pathway analysis of DEGs in three comparisons. A, B, and C represent the pathway analyses of DEGs in I0 vs. I2, I2 vs. I4, and I4 vs. I6 comparisons, respectively.

To obtain an insight into the similarities and differences in the differentiation of intramuscular preadipocytes at different stages, the mRNAs differentially expressed in three comparisons (I0 vs. I2, I2 vs. I4, and I4 vs. I6) were subjected to GO (biological process) and pathway analyses. The top three biological process terms were cell adhesion, biological adhesion, and developmental process at the I0–I2 stage (day 0 to day 2 of differentiation); cell cycle phase, cell cycle, and mitotic cell cycle at the I2–I4 stage (day 2 to day 4 of differentiation); and cell cycle process, cell cycle phase, and M phase at the I4–I6 stage (day 4 to day 6 of differentiation) (Fig 6, S63Appendix). We found that nine terms were common between the I2 vs. I4 and I4 vs. I6 comparisons, while only one common term was found between the I0 vs. I2 and I4 vs. I6 comparisons. No common term was found between the I0 vs. I2 and I2 vs. I4 comparisons. Interestingly, we identified several biological process terms related to cell differentiation, including cell differentiation, fat cell differentiation, and brown fat cell differentiation. The pathway results showed that 10, 12, and 10 pathways were significantly enriched for the I0–I2, I2–I4, and I4–I6 comparisons, respectively (Fig 7; S4 Table). The Fanconi anemia pathway, Extracellular Matrix (ECM)–receptor interaction, and malaria were the top three pathways for the I0–I2 comparison; the Fanconi anemia pathway, alanine, aspartate, and glutamate metabolism, and DNA replication were the top three pathways for the I2–I4 comparison; while cell cycle, PPAR signaling pathway, and pathways in cancer were the top three pathways for the I4–I6 comparison. Interestingly, we found that the PPAR and p53 signaling pathways, which have been proven to play key roles in the differentiation of preadipocytes, were involved in the entire differentiation process of intramuscular preadipocytes in our study (S4 Appendix). A large number of differentially expressed genes were assigned to the PPAR and p53 pathways, including *FABP4*, *PPARG*, *p53R2*, and *APOA1*.

### Co-expression network construction and module detection

lncRNAs exert their biological functions by regulating target mRNAs. Co-expression network analysis could help us to predict the target mRNAs of lncRNAs by detecting their similar expression patterns, so the WGCNA R software package was used here for this purpose. Before this analysis, differentially expressed genes with a low expression level (FPKM ≤0.05) in more than one sample in the same group were removed. Finally, 2,510 differentially expressed genes (379 lncRNAs and 2,131 mRNAs) were retained and subjected to co-expression analysis (S5 Table). The node and edge files correspond to gene expression profiles and pairwise correlations between gene expressions, respectively. A total of 419 mRNAs were identified to have common expression patterns with 94 lncRNAs, which might be target genes of lncRNAs (S6 Table).

**Fig 8:**
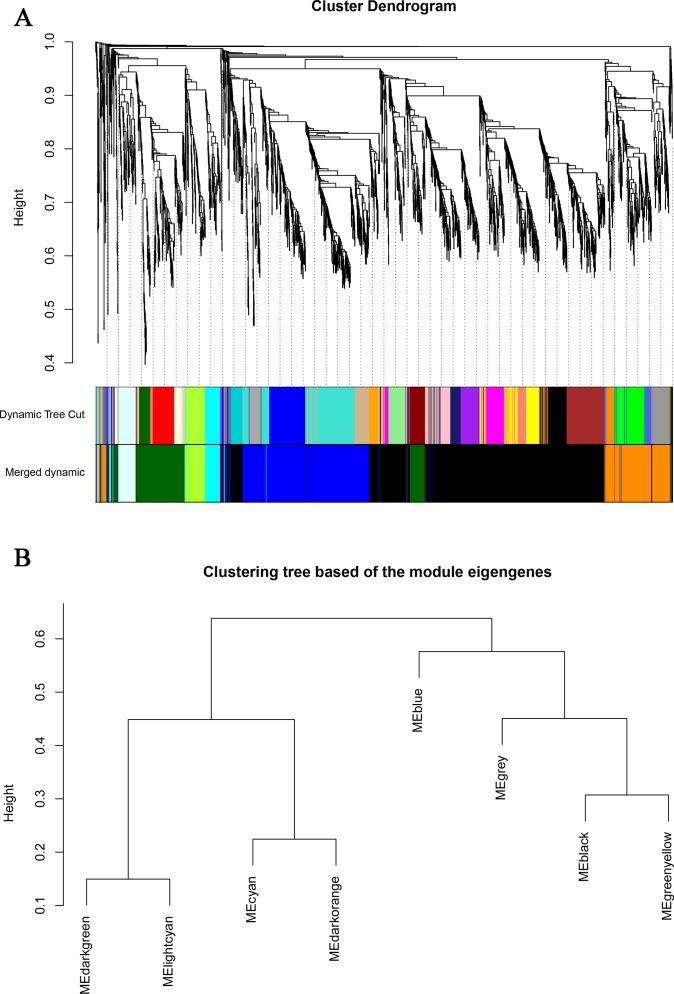
Visualization of module detection and clustering. A) Hierarchical cluster tree (average linkage, dissTOM) of the 2,510 genes. The color bands provide a simple visual comparison of module assignments (branch cuttings) based on the dynamic hybrid branch cutting method. B) Clustering of modules based on eigengenes.

WGCNA can be used to find clusters (modules) of highly correlated genes, to summarize such clusters using the module eigengene (ME) or an intramodular hub gene, and to relate modules to one another and to external sample traits [32]. In our study, 2,510 DEGs were used to identify groups of co-expressed genes, termed “modules”. Each module is assigned a unique color label underneath the cluster tree [34]. Eight modules were identified, ranging in size from 11 genes for the grey module to 1,052 genes for the dark grey module (Fig 8A, S7 Table). The co-expression modules could not exist independently; instead, they formed a meta-network. To explore and identify the correlations among the modules, 8 modules were subjected to clustering analysis based on their eigengenes. The results showed that these 8 modules could be classified into three groups: the first included dark green and light cyan; the second included cyan and dark orange; and the third included grey, black, green yellow, and blue (Fig 8B). Modules classified into the same group are suggested to have the same or similar functions and regulatory mechanisms.

### Stage-specific module identification

**Fig 9:**
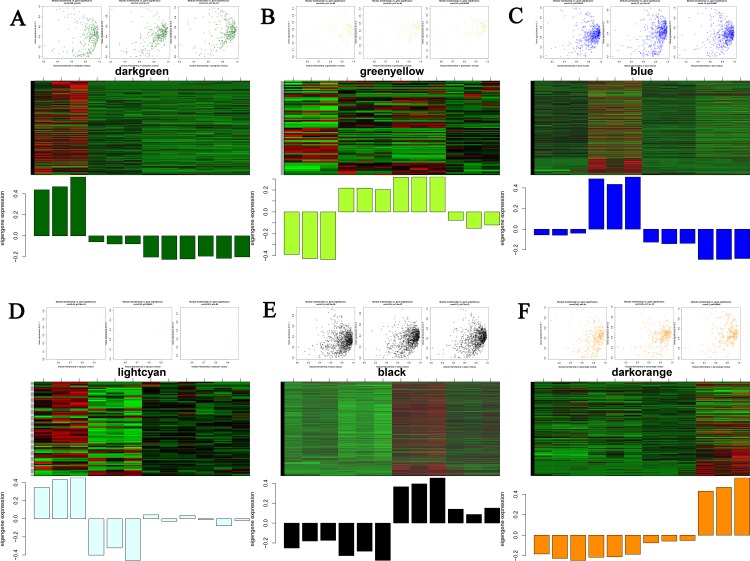
Visualization of GS vs. MM and expression level in stage-specific modules. The scatter plot shows the distribution of the GS and MM of genes in stage-specific modules related to one sample. The *P*-value represents the significance of a module related to one stage. The heatmap and bar plot correspond to the expression level (ME) of genes in a stage-specific module in the 12 samples (from left to right: I0-1, I0-2, I0-3, I2-1, I2-2, I2-3, I4-1, I4-2, I4-3, I6-1, I6-2, and I6-3).

**Fig 10:**
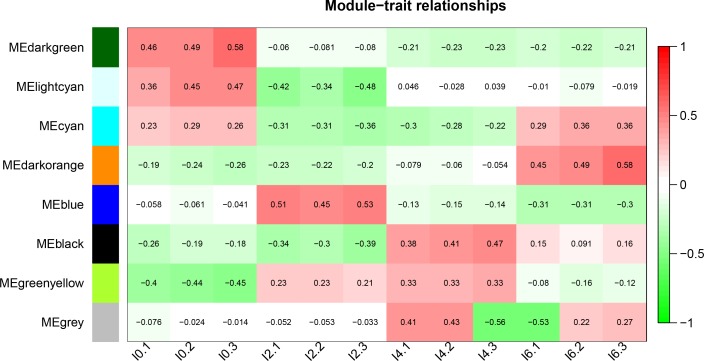
Correlation between modules and differentiation stage. The color, ranging from green through white to red, indicates negative to positive correlation.

To explore stage-specific modules during chicken intramuscular preadipocyte differentiation, we calculated the GS and MM of all genes within a module (Fig 9). GS was defined as (the absolute value of) the correlation between the gene and the differentiation stage. MM was defined as the correlation of the module eigengene and the gene expression profile. A strong correlation between GS and MM (*P*<0.05) illustrates that genes highly significantly associated with a trait are often also the most important (central) elements of modules associated with that trait. In addition, we used ME to represent the expression level of genes in each module. The correlations between ME and the stage of differentiation were analyzed (Fig 10). Finally, we identified six stage-specific modules (average module–trait relationship >0.4 and *P*<0.05), among which the green yellow and light cyan modules were negatively correlated with the I0 and I2 stages. The expression levels (ME) of genes in the green yellow and light cyan modules were predominantly downregulated at days 0 and 2 of differentiation. In contrast, the dark green, blue, black, and dark orange modules were positively correlated with the I0, I2, I4, and I6 stages, respectively, with the expression of genes in those modules being predominantly upregulated at days 0, 2, 4, and 6 of differentiation.

### Identification and visualization of hub genes

**Fig 11:**
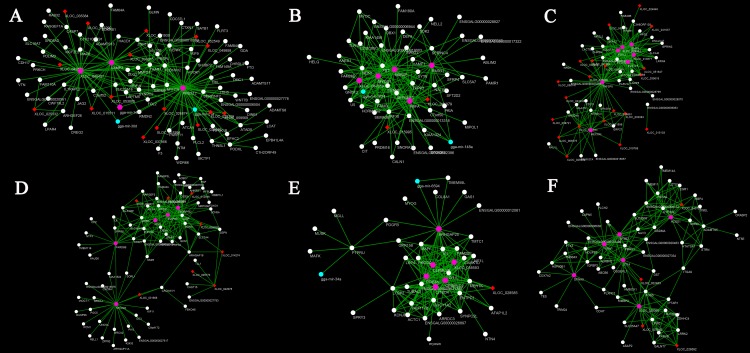
Visualization of hub genes in stage-specific modules. A–F represent the visualization of the top 200 connections of the top 150 hub genes for black, blue, dark green, dark orange, green yellow, and light cyan modules. Red nodes represent lncRNAs, white nodes represent mRNAs and cyan nodes represent miRNAs. Pink nodes represent highly connected genes in stage-specific modules.

To identify genes that are central and highly connected in the stage-specific modules, we conducted hub gene identification analysis using GS and MM measures in the WGCNA package. Hub genes are significantly associated with one or more stages of preadipocyte differentiation. Genes with GS >0.4 and MM >0.8 were identified as potential hub genes for each module (S5 Appendix). Given that we set up three biological replicates at each stage, common potential hub genes were screened using a Venn diagram among the three replicates at the same differentiation stage. The common potential hub genes were finally identified as hub genes related to the differentiation of intramuscular preadipocytes (S8 Table). Furthermore, to explore the connections among hub genes, we analyzed the top 200 connections of the top 150 potential hub genes for each stage-specific module and visualized them in Cytoscape (Fig 11). Although there are well-studied genes related to preadipocyte differentiation, such as *IGFBP2*, *Ex-FABP*, *MYOD1*, and *BMP3*, many of these genes are reported here for the first time in the differentiation of intramuscular preadipocytes in chicken. For example, ENSGALG00000023180 (mean FPKM = 39.91) and ENSGALG00000027887 (mean FPKM = 34.22) were highly correlated, expressed, and unannotated hub genes in chicken intramuscular preadipocytes. Furthermore, many highly correlated lncRNAs were identified, including XLOC_040491, XLOC_029050, and XLOC_057619, which might play key roles in their specific stages of differentiation (Table 2).

**Table 2:** Highly connected hub genes in chicken stage-specific modules.

Stage-specific module	Stage	Hub genes
Dark green	Day 0	*XLOC_013577*, *XLOC_029044*, *BMP3*, *KLHL3*, *ELOVL7*
Green yellow		*XLOC_058593*, *ARHGAP25*, *PDE3A*, *CITED4*
Blue	Day 2	*XLOC_057619*, *MYOD1*, *MXRA5*, *RBFA*, *RASD1*
Light cyan		*XLOC_029050*, *ST14*, *DFNA5*, *VWA1*, *ACSS1*
Black	Day 4	*XLOC_040491 LAMP3*, *PRSS12*, *gga-mir-146b*
Dark orange	Day 6	*C6ORF162*, *DIAPH3*, *ELN*, *PARD6B*, *MYOCD*

### Validation of DEGs by qRT-PCR

**Fig 12:**
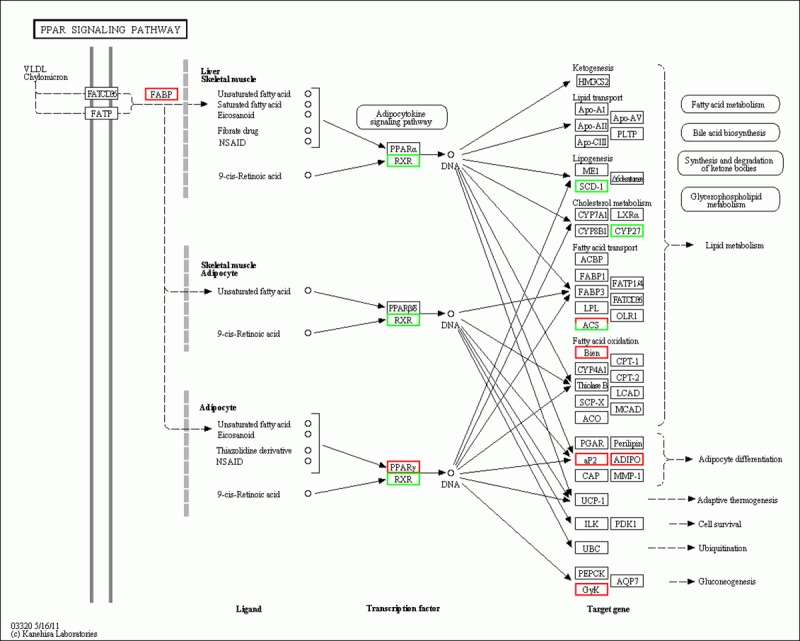
Differentially expressed genes involved in PPAR signaling pathway. Red and green represent upregulated and downregulated genes.

**Fig 13:**
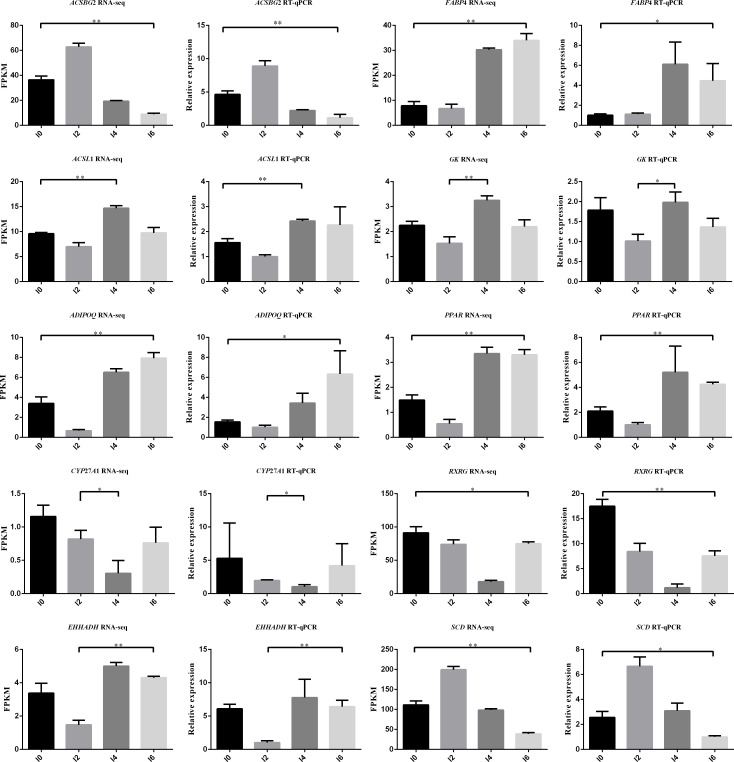
Validation of differentially expressed genes in the PPAR signaling pathway. *P<0.05, **P<0.01.

**Fig 14:**
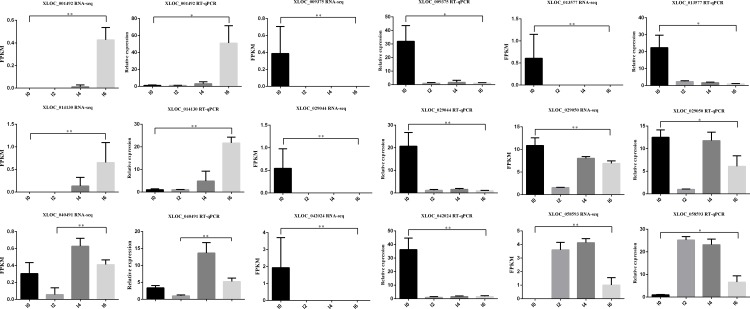
Validation of differentially expressed lncRNAs by qRT-PCR. *P<0.05, **P<0.01.

Quantitative real-time PCR (qRT-PCR) was carried out to validate the differentially expressed genes involved in the PPAR signaling pathway (*ACSL1*, *CYP27A1*, *A-FABP*, *SCD-F*, *GK*, *ACSBG2*, *ADIPOQ*, *PPARG*, *EHHADH*, and *RXRG*) (Fig 12). In addition, 9 hub lncRNAs, including 5 highly connected lncRNAs were validated by qRT-PCR. We used the same 12 cell samples as were used in the RNA-seq for qRT-PCR validation. The qRT-PCR results for all of the DEGs were analyzed statistically using t-test. The results showed that the expression patterns of these 10 genes and 9 lncRNAs were in excellent agreement with the RNA-seq results (Fig 13, Fig 14, S9 Table).

## Discussion

In the last 60 years, the selection of important economic traits has been the focus and significant genetic improvements have been achieved [35]. However, the quality and flavor of chicken have decreased as a result of genetic selection for faster growth and increased feed conversion efficiency. Studies have shown that IMF content is associated with multiple meat quality characteristics, such as tenderness, juiciness, flavor, and water holding capacity, in chicken, pork, and beef. It was reported that preadipocyte differentiation is a key factor affecting IMF deposition [36]. However, to date, only a few studies have identified genes that play important roles in porcine intramuscular preadipocyte differentiation, such as *FABP1* [37], *FABP4*, *GPR39* [7], and *CD36* [38]. The mechanisms by which intramuscular preadipocyte differentiation and fat deposition are regulated have also remained unclear in chicken.

lncRNA is a kind of noncoding RNA longer than 200 nt, which has attracted substantial attention in the last few years. Studies have shown that lncRNAs regulate metabolic tissue development and function, including adipogenesis, hepatic lipid metabolism, islet function, and energy balance [39–43]. Despite the fact that many studies have indicated the importance of lncRNAs in different tissues, little is known about their biological function in chicken fat deposition, especially in the differentiation of chicken intramuscular preadipocytes. To the best of our knowledge, our study is the first to screen for lncRNAs and mRNAs regulating chicken intramuscular preadipocyte differentiation by sequencing and annotating the transcriptome of intramuscular preadipocytes at different stages. After quality control, an average of 16.39 Gb of clean reads were obtained per intramuscular preadipocyte sample. A total of 997,816,749 reads were successfully mapped to the chicken reference genome assembly. As the first study of lncRNAs in intramuscular preadipocytes of chicken, we identified 25,435 novel lncRNAs. The results showed that most lncRNAs contained only one exon. The ORF length of lncRNAs mainly ranged from 20 to 100 bp, while the sequence length was mainly distributed in the range of 200–400 nt. Our results indicated that the predicted lncRNAs were shorter and contained fewer exons than mRNAs, in agreement with previous studies [16, 44].

Many studies have shown that the expression of lncRNAs can regulate the expression of neighboring mRNAs [16, 45, 46]. Based on this, we searched for coding genes 100 kb upstream and downstream of lncRNAs as cis target genes to predict the function of the lncRNAs. We also searched for mRNAs that could undertake complementary binding to antisense lncRNAs as target genes. Consequently, we found that many target genes of differentially expressed lncRNAs were also differentially expressed. This suggested that lncRNAs may function through neighboring or complementary target genes, which can play critical roles in the differentiation of chicken intramuscular preadipocytes. For example, *IGFBP2* is a target gene upstream of the differentially expressed lncRNAs XLOC_054724 and XLOC_054725, and has been reported to regulate the differentiation of preadipocytes [47, 48]. These findings suggest that XLOC_054724 and XLOC_054725 are involved in the differentiation of intramuscular preadipocytes by affecting the expression of *IGFBP2*.

To identify genes associated with the differentiation of preadipocytes in chicken, we compared the transcriptome-wide gene expression profiles between the libraries of the four differentiation stages. In total, 4,698 differentially expressed lncRNAs and 2,735 differentially expressed mRNAs were obtained by pairwise comparisons (I0 vs. I2, I0 vs. I4, I0 vs. I6, I2 vs. I4, I2 vs. I6, and I4 vs. I6) of samples collected from preadipocytes at days 0, 2, 4, and 6 of differentiation. A total of 3,200 differentially expressed lncRNAs and 2,087 differentially expressed mRNAs were obtained by pairwise comparisons (I0 vs. I2, I2 vs. I4, I4 vs. I6) of the same samples. Sixty-four genes (7 lncRNAs and 57 mRNAs) were differentially expressed in the entire differentiation process, suggesting their importance in the differentiation of intramuscular preadipocytes. An example of these is *MYOD1*, a member of the *MyoD* gene family, which has been reported to promote brown adipose tissue development [49].

To explore the similarities and differences of different differentiation stages, 1,608 genes known to encode proteins that were differentially expressed in three comparisons (I0 vs. I2, I2 vs. I4, and I4 vs. I6) were subjected to GO and pathway analyses. We found that nine terms were common between the I2 vs. I4 and I4 vs. I6 comparisons, while only one common term was found between the I0 vs. I2 and I4 vs. I6 comparisons. No common term was found between the I0 vs. I2 and I2 vs. I4 comparisons. We also performed clustering analysis on 12 samples with the expression of all DEGs at different differentiation stage (S1 Fig). The results indicated a strong correlation between preadipocytes at days 4 and 6 of differentiation. The above findings suggest that the changes of gene expression in early differentiation stages (I0 and I2) are much more dramatic than those in later differentiation stages (I4 and I6). To the best of our knowledge, only a few pathways involved in intramuscular preadipocyte differentiation have been validated to date, including the PI3K/AKT cell signaling pathway [7], BMP-Smad signaling pathway [50], and C/EBPβ and PI3K/GSK3β signaling pathway [51]. Building on this previous work, we have identified more than 60 pathways the components of which were significantly enriched in the differentiation process. Several pathways involved in preadipocyte differentiation were previously identified, including the PPAR signaling pathway [52], TGF-β signaling pathway [53], and arachidonic acid metabolism [54]. However, for most pathways identified here, their involvement in the preadipocyte differentiation process is being reported for the first time. Interestingly, in the pathway analysis, we found that the components of two pathways, the PPAR and p53 signaling pathways, which have been reported to be involved in the differentiation of preadipocytes, were enriched in the entire process of differentiation of intramuscular preadipocytes [55–57]. A large number of DEGs were assigned to these two pathways, including the well-studied genes *PPARG*, *A-FABP*, *CDC2*, and *CCNB2*. Tannic acid and 4-O-methylascochlorin were shown to suppress the differentiation of 3T3-L1 preadipocytes by inhibiting the expression of the PPARG gene [58, 59]. A-FABP is considered as a candidate for intramuscular fat accretion and is strongly related to the development and accretion of intramuscular fat in pig [60]. The overexpression of the cattle A-FABP gene was also found to promote fat deposition in the skeletal muscle of transgenic mice [61]. In addition, the adipokine chemerin promotes lipolysis in mature adipocytes and induces adipogenesis during preadipocyte re-differentiation by upregulating the expression of A-FABP [62]. CDC2, also known as CDK1, regulates the alternative splicing and adipogenesis in 3T3-L1 preadipocytes [63]. It also regulates the differentiation of multiple cells, such as terminal lens fiber cells [64], T cells [65], chondrocytes [66], and embryonic stem cells [67]. These findings suggest that the PPAR and p53 signaling pathways play critical roles in the differentiation of intramuscular preadipocytes in chicken. Ten differentially expressed genes involved in the PPAR signaling pathway from the I0 vs. I2 comparison were validated by qRT-PCR and the results were in excellent agreement with the RNA-seq findings. This suggests that our RNA-seq findings are reliable.

Studies have shown that genes and the proteins that they encode carry out cellular processes in the context of functional modules and are related to each other through a complex network of interactions. Understanding an individual gene or protein’s network properties within such networks may prove to be as important as understanding its functions in isolation [68–70]. Therefore, the primary emphasis in our study was on constructing a coexpression network and detecting modules related to intramuscular preadipocyte differentiation. In this work, integrated WGCNA was used to construct a co-expression network and detect modules with 2,510 differentially expressed genes (lncRNAs and mRNAs) [71]. Using WGCNA, we identified 8 modules, six of which were stage-specific [72]. This means that those modules included genes that were down- or upregulated in a particular differentiation stage and can be used to present this particular stage [73]. The green yellow and light cyan modules were negatively correlated with I0 and I2 stages, indicating that the expression levels (ME) of genes in these two modules were predominantly downregulated at days 0 and 2 of differentiation. Instead, the dark green, blue, black, and dark orange modules were positively correlated with the I0, I2, I4, and I6 stages, with the expression of the genes in these modules being predominantly upregulated at days 0, 2, 4, and 6 of differentiation. The presence of genes together in one module suggests that they are involved in a common network of biological processes and functions.

To date, many genes have been reported to regulate the differentiation of intramuscular preadipocytes. However, no study has been conducted on the roles of lncRNAs in intramuscular preadipocyte differentiation. The molecular and cellular mechanisms regulating chicken intramuscular preadipocyte differentiation are thus still poorly understood. Here, using the criteria of GS >0.4 and MM >0.8, we identified more than 300 hub genes from six stage-specific modules. In the day 0 stage-specific module (dark green and green yellow), 56 hub genes, including the well-studied gene *BMP2*, were identified. Its product, bone morphogenetic protein 2, is a member of the transforming growth factor (TGF)-β superfamily involved in multiple steps of differentiation [74]. *BMP2* has been reported to induce adipocytic differentiation in 3T3-F442A [75] and STS-L1 [76] cells. *BMP2* controls adipocytic differentiation by inducing and upregulating PPARγ via Smad and p38 kinase signaling [77]. A total of 155 hub genes were identified in the day 2 stage-specific modules (blue and lightcyan), among which *KLF2* has been well studied and reported to inhibit chicken adipogenesis through the inhibition of *PPARγ* and *C/EBPα* expression [78]. In the day 4 stage-specific module (black), 51 hub genes were identified, including the well-known gene *BMP4*. *BMP4* downregulates *PDGFRβ* by stimulating lysosome-dependent degradation, which efficiently initiates adipogenic differentiation [79]. *BMP4* is an integral feedback regulator of both white and beige adipogenic commitment and differentiation [80]. In the day 6 stage-specific module (darkorange), 55 hub genes were identified, including the *TGFB3* gene, which can inhibit adipogenesis and interacts with the transcription factor paired-related homeobox 1 (*Prrx1*) [81]. To explore the connections between hub genes, we analyzed the top 200 connections of the top 150 potential hub genes for each stage-specific module and visualized them in Cytoscape. We identified a number of highly connected lncRNAs and mRNAs in the six stage-specific modules, including *XLOC_058593*, *XLOC_013577*, and *MYOD1* (Table 2). To the best of our knowledge, the involvement of all of the identified hub lncRNAs in the differentiation of intramuscular preadipocytes is reported for the first time in this paper. The functions of most identified hub coding genes have not been studied or annotated in the differentiation of intramuscular preadipocytes in chicken. Collectively, our findings of all of the hub genes (lncRNAs and mRNAs) provide a valuable resource for further study of the molecular mechanisms of intramuscular preadipocyte differentiation. However, these predicted functions of lncRNAs require experimental verification.

## Supporting information

S1 Appendix Sequences of all lncRNAs in intramuscular preadipocytes. (ZIP)

S2 Appendix all differentially lncRNAs and mRNAs. (ZIP)

S3 Appendix Biological process analysis of DEGs in three comparisons (I0 vs I2, I2 vs I4 and I4 vs I6). (ZIP)

S4 Appendix Common pathways involved in the entire differentiation process of intramuscular preadipocytes. (ZIP)

S5 Appendix Potential hub genes for stage-specific modules. (ZIP)

S1 Fig The correlation between 12 samples. (TIF)

S1 Table Primers of differentially expressed mRNAs and lncRNAs. (DOCX)

S2 Table Common DEGs anmong three comparisons (I0 vs. I2, I2 vs. I4, and I4 vs. I6). (XLSX)

S3 Table Genes involved in 11 clusters. (XLXX)

S4 Table Significant enriched pathways of all DEGS. (XLSX)

S5 Table Differentially expressed genes subjected to WGCNA analysis. (CSV)

S6 Table Co-expressed lncRNAs and mRNAs. (XLSX)

S7 Table Genes in eleven modules. (XLSX)

S8 Table Hub genes of stage-specific modules. (XLSX)

S9 Table Validation of RNA-seq using RT-qPCR. (XLSX)
