# Supplementary figures and images for: Analysis of long noncoding RNA and mRNA using RNA sequencing during the differentiation of intramuscular preadipocytes in chicken
Source: PLoS One. 2017 Feb 15;12(2):e0172389. doi: 10.1371/journal.pone.0172389 (PMC5310915; doi:10.1371/journal.pone.0172389)

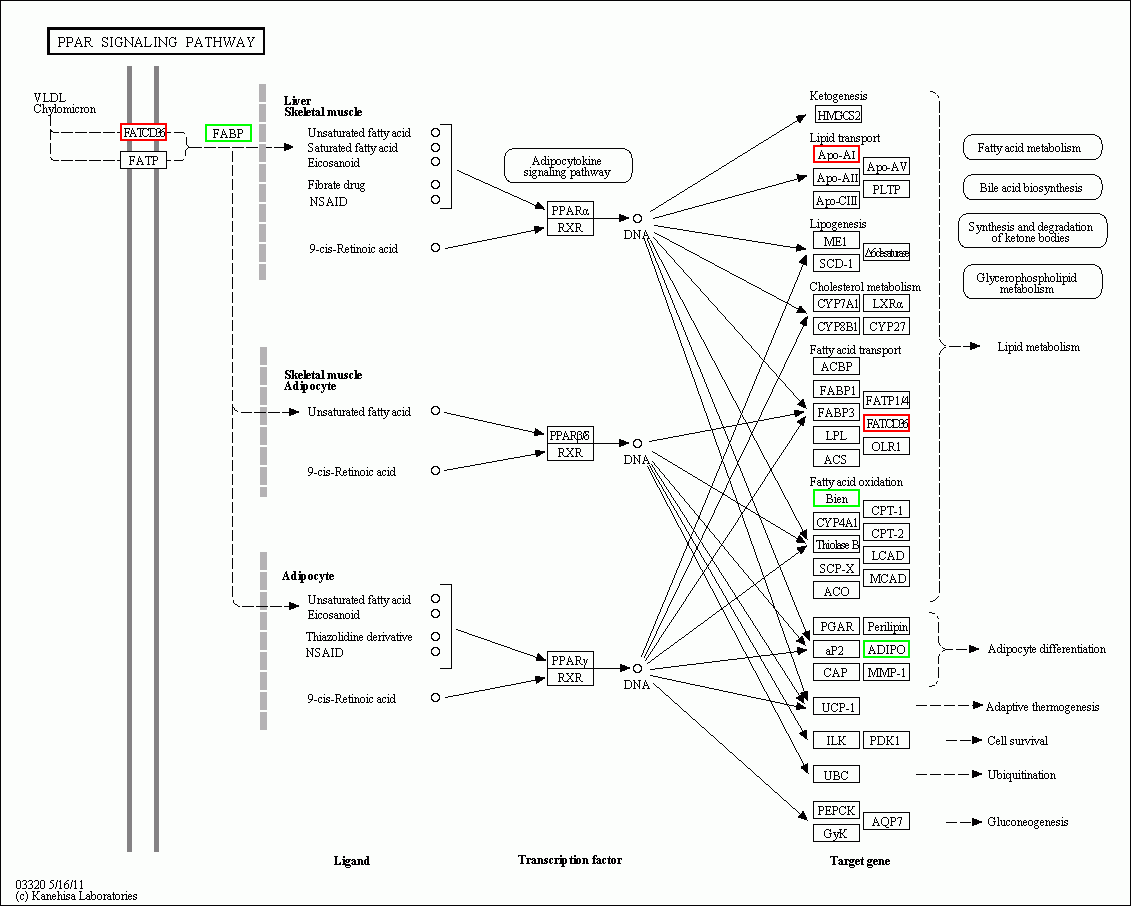

Supplement: S4 Appendix — (ZIP) [file pone.0172389.s004.zip › I0-I2 PPAR pathway.png]

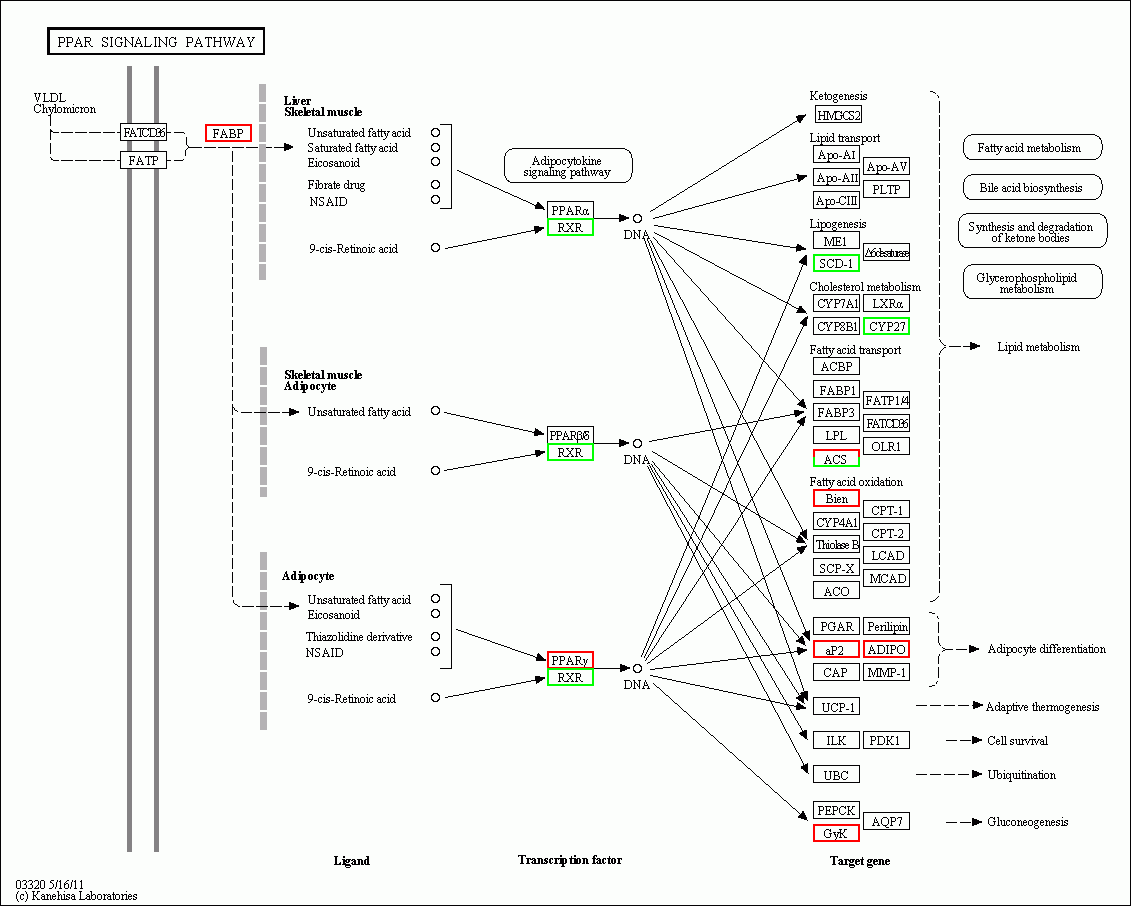

Supplement: S4 Appendix — (ZIP) [file pone.0172389.s004.zip › I2-I4 PPAR pathway.png]

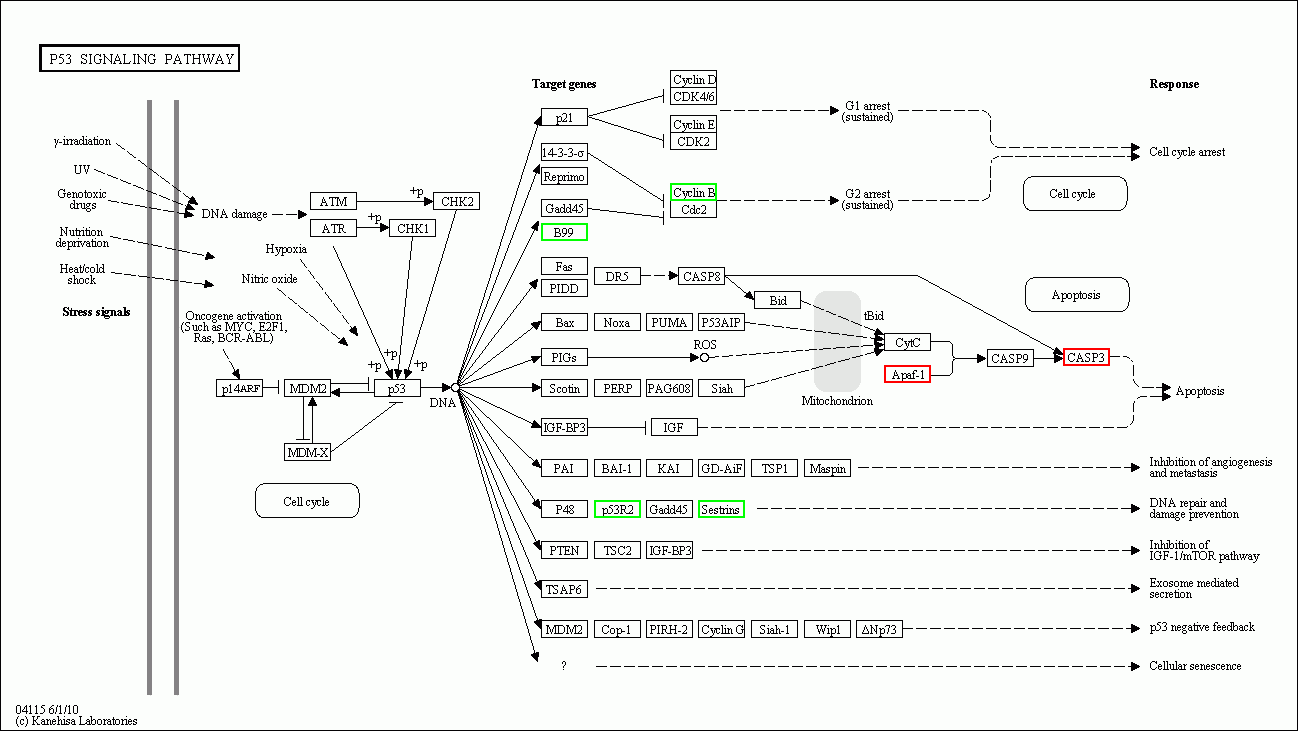

Supplement: S4 Appendix — (ZIP) [file pone.0172389.s004.zip › I2-I4 p53 pathway.png]

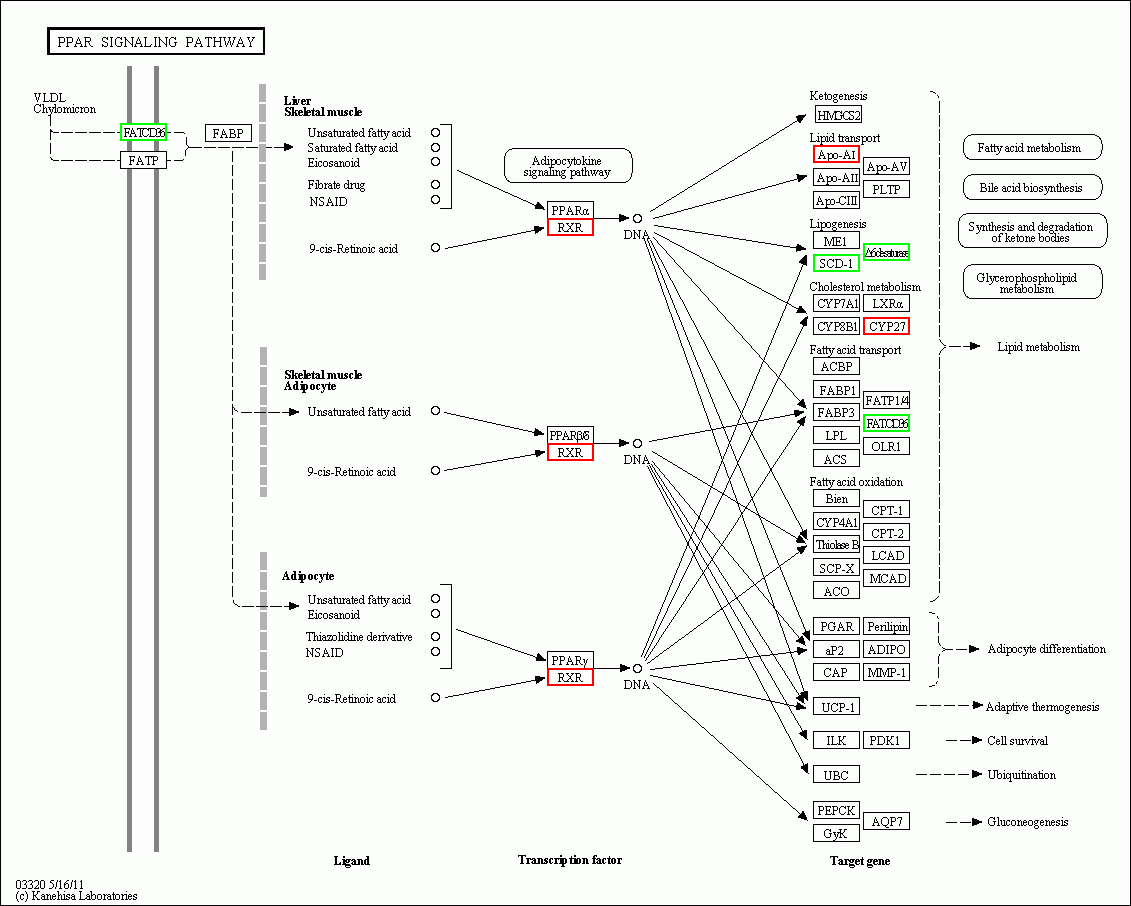

Supplement: S4 Appendix — (ZIP) [file pone.0172389.s004.zip › I4-I6 PPAR pathway.png]

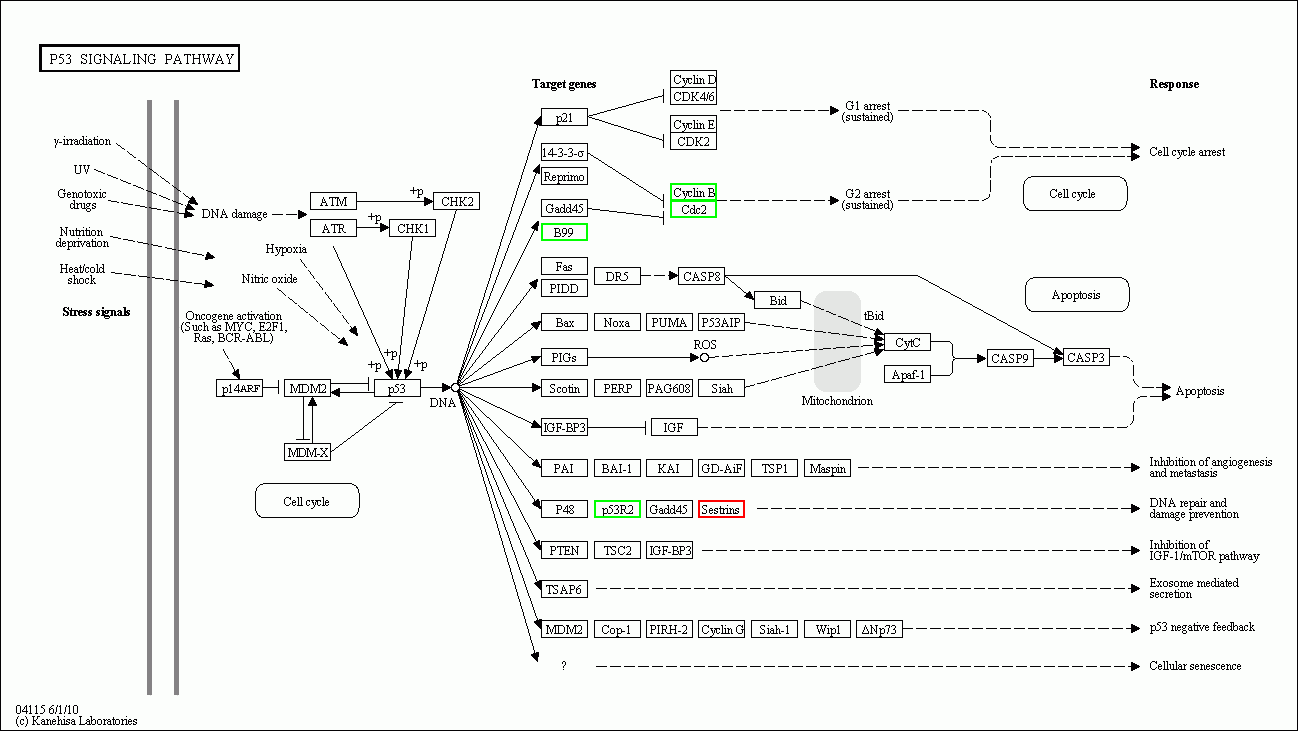

Supplement: S4 Appendix — (ZIP) [file pone.0172389.s004.zip › I4-I6 p53 pathway.png]

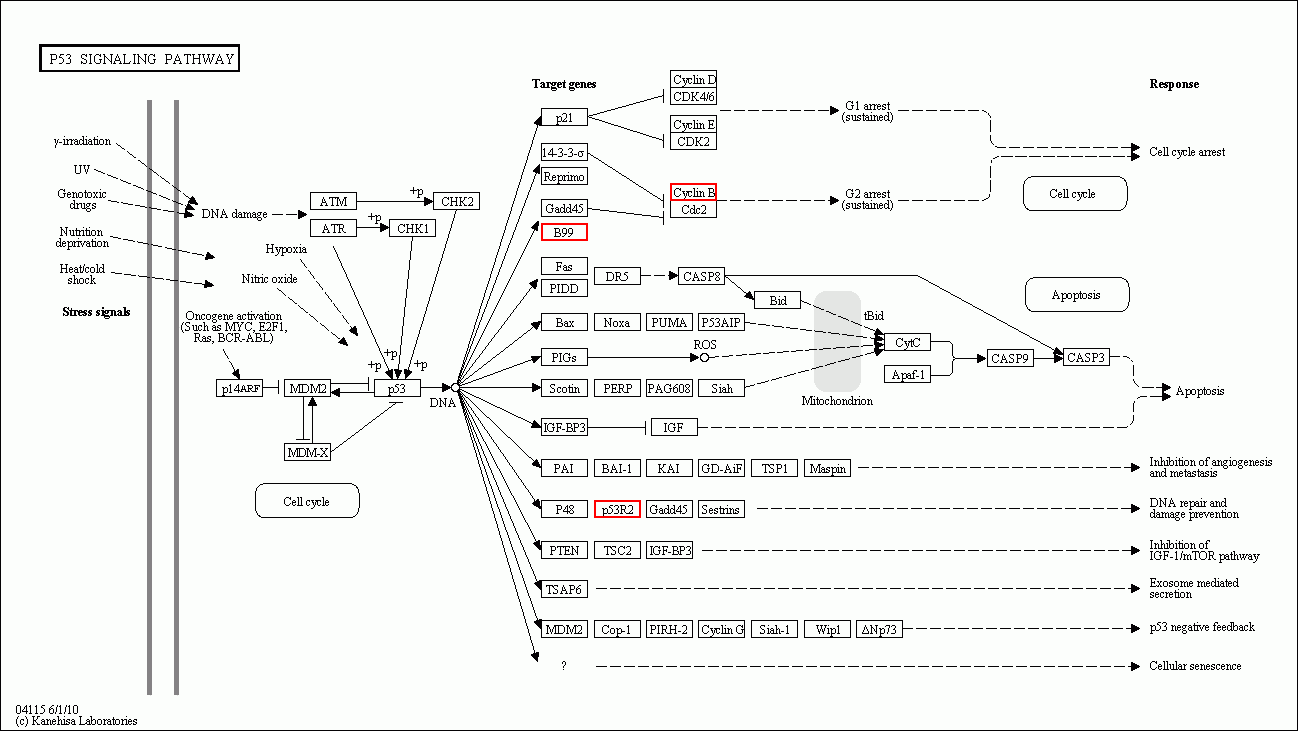

Supplement: S4 Appendix — (ZIP) [file pone.0172389.s004.zip › I0-I2 P53 pathway.png]

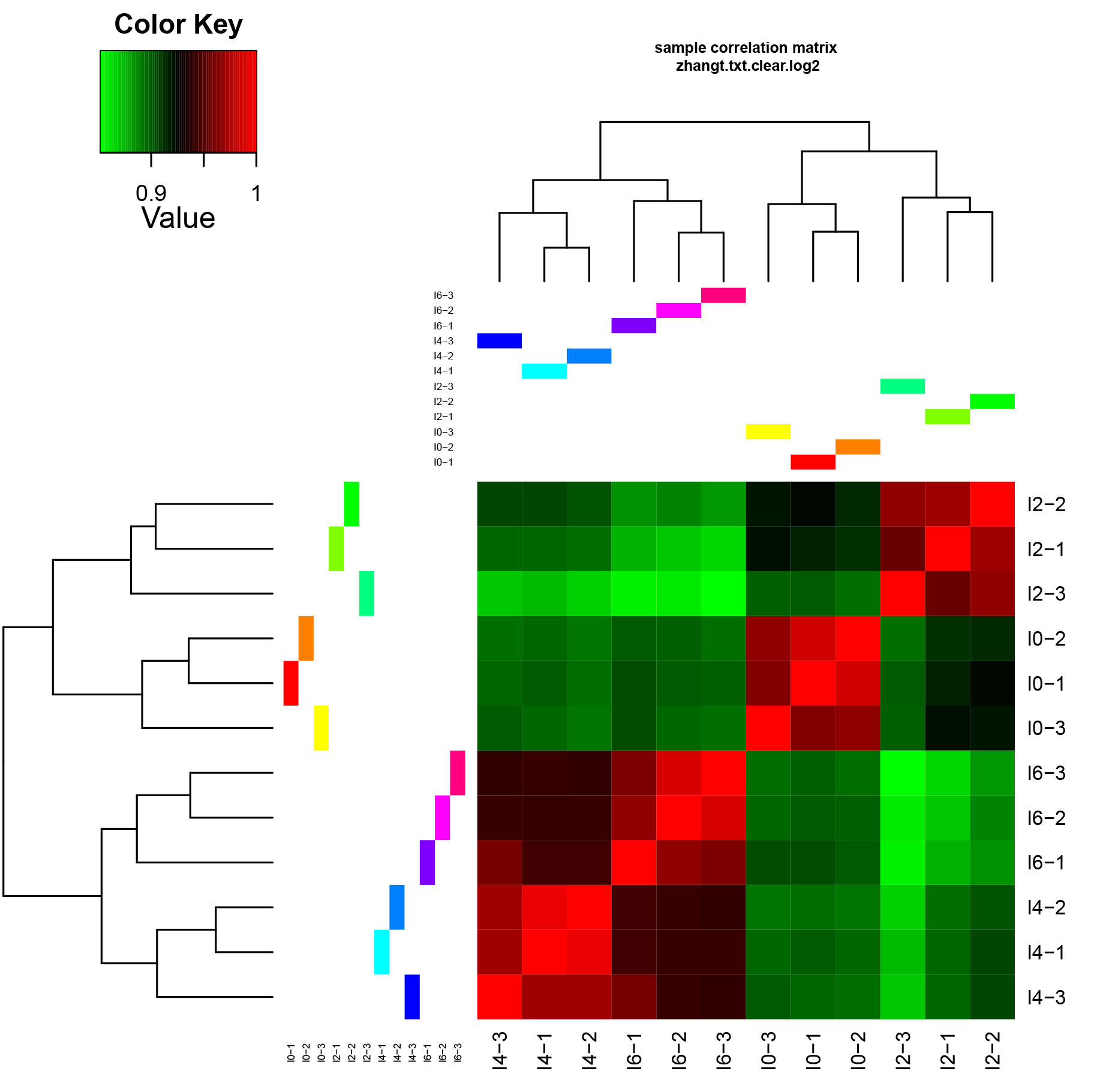

Supplement: S1 Fig — (TIF) [file pone.0172389.s006.tif]
