# Supplementary material for: Analysis of long noncoding RNA and mRNA using RNA sequencing during the differentiation of intramuscular preadipocytes in chicken
Source: PLoS One. 2017 Feb 15;12(2):e0172389. doi: 10.1371/journal.pone.0172389 (PMC5310915; doi:10.1371/journal.pone.0172389)
Supplement: S1 Table — (DOCX) [file pone.0172389.s007.docx]

S1 Table. Primers of differentially expressed mRNAs and lncRNAs

| Genename | Forward primer | Reverse primer | product length | TM | Accession number |
| --- | --- | --- | --- | --- | --- |
| *FABP4* | GCCTGACAAAATGTGCGACC | ATTAGGCTTGGCCACACCAG | 130 | 60 | NM_204290.1 |
| *PPARG* | TTTTCAAGCATTTCTTCACCACACT | GGAGAAGGAGGCTCCATTTTGA | 131 | 60 | NM_001001460.1 |
| *RXRG* | ATTCCGGTGAGAGGAGACGG | ATGATGGGCTCACAGACGTG | 150 | 60 | NW_003763828.1 |
| *ADIPOQ* | CTCGCTGCTATTAAGGCCCC | CAACATCCAGGAGGCTACACT | 126 | 60 | NM_206991.1 |
| *GK* | AGCTTTGACAGGTGTGCCA | GGCCTGTATTGCACAGCAAG | 134 | 60 | XM_003640511.3 |
| *EHHADH* | GGTTTTCCCTTGCACAGCAG | CTTTGCCCGAAGCATGGAAG | 103 | 60 | XM_422690.5 |
| *ACSL1* | GACCGGTCTGTGCGTTGTT | ACAAGTCGTGTGCTTGCATC | 87 | 60 | NM_001012578.1 |
| *ACSBG2* | CTGTTCCCGCGTCGTG | TCTGACTCGCACAGCATCATC | 70 | 60 | NM_001012846.2 |
| *SCD* | CTGTGCTTCGTGTTGAGTGC | CGGGCCCACTCATAGATGTC | 149 | 60 | NM_204890.1 |
| *CYP27A1* | TCAGGACTTTCGTCTGGCTC | CCAGATGGGGCCATAGATGC | 92 | 60 | XM_422056.5 |
| XLOC_001492 | CTGTCCAAACGCACAGCATC | AGGGAGGTCCTTCTTGTTTTGG | 142 | 60 | XLOC_001492 |
| XLOC_013577 | GGAGAAACACCTTTGCCCCT | TTCGTGAAGGGCAAGTCACA | 78 | 60 | XLOC_013577 |
| XLOC_014130 | CATGGCGGATGACTCTTGCT | TGGCCCATTTCCTGCATACT | 80 | 60 | XLOC_014130 |
| XLOC_058593 | TCTCTGGAACATCATCAGCCA | GCAGTCGGTTGCAGGGAATA | 102 | 60 | XLOC_058593 |
| XLOC_009375 | CGGCTAAGCTTCACTTTGGA | ACAAAAGGCAGAGCACTTCG | 133 | 60 | XLOC_009375 |
| XLOC_029044 | TCTGCCAGGGTTTGCAAGAT | AGCCCTTGAAAGCATACCCC | 110 | 60 | XLOC_029044 |
| XLOC_057619 | CCCGGTGGTAGGAAAGAACC | TCCGTCCTGGCTCAGTTTTC | 110 | 60 | XLOC_057619 |
| XLOC_029050 | AGATGTGTGGGAGGATCCGA | ATTGCCAGGGCTTGAAGGTT | 104 | 60 | XLOC_029050 |
| XLOC_040491 | AGGGACACCTCCTTCTAGACC | AGTTGGCCTCACCTGTGGAA | 114 | 60 | XLOC_040491 |
